# First case report of *Providencia Rettgeri* neonatal sepsis

**DOI:** 10.1186/s13104-017-2866-4

**Published:** 2017-10-30

**Authors:** Deepak Sharma, Pradeep Sharma, Priyanka Soni

**Affiliations:** 1 DNB Neonatology, NEOCLINIC, TN Mishra Marg Everest Vihar Nirman Nagar, Jaipur, Rajasthan India; 2 Department of Medicine, Mahatma Gandhi Medical College, Jaipur, Rajasthan India; 3 Department of Microbiology, J.L.N Medical College, Ajmer, Rajasthan India

**Keywords:** *Providencia rettgeri*, Neonate, Late onset sepsis

## Abstract

**Background:**

Providencia are gram negative motile rods and is a member of the Enterobacteriaceae family. It consists of five species, namely *Providencia alcalifaciens*, *Providencia rustigianii*, *Providencia stuartii*, *Providencia rettgeri* and *Providencia heimbachae*. These are opportunistic pathogens and leads to infections in immunocompromised host. Providencia rettgeri has been associated with the nosocomial infections of the urinary tract and infections of wounds, burns and blood. *Providencia rettgeri* is very rare cause of neonatal sepsis and we report first case of neonatal late onset sepsis secondary to it.

**Case presentation:**

A term male infant presented on day 4 of post-natal life with the complaint of decreased appetite, fast respiration and lethargy. The clinical examination showed features of sepsis and shock with chest radiogram showing pneumonia. The infant was started on invasive ventilation, intravenous fluids, antibiotic and inotropes. The blood culture was suggestive of multi-drug resistant *P. rettgeri*. The antibiotics were changed according to organism antibiotic susceptibility pattern and infant gradually improved and was discharged successfully.

**Conclusion:**

*Providencia rettgeri* is a very rare organism to cause neonatal sepsis. The management involves early diagnosis, treatment with appropriate antibiotics and finding the source of infection.

## Background

The bacteria of the genus *Providencia* have been included in the tribe Proteeae of family Enterobacteriaceae and consist of five species. The peculiar ability to deaminate oxidatively specific amino acids to their corresponding keto acid and ammonia, makes them different from other members of the family Enterobacteriaceae. The different species are *Providencia alcalifaciens*, *Providencia rustigianii*, *Providencia stuartii*, *Providencia rettgeri* and *Providencia heimbachae* [1]. *Providencia rettgeri* was discovered by Rettger (1909) from a cholera like epidemic in chickens. The other properties discovered by investigators were of fermenting mannitol and being anaerobic [2]. To our knowledge, we report the first case of *Providencia rettgeri* infection in a neonate leading to late onset neonatal sepsis. The organism was multi-drug resistant and the infant was treated with antibiotics as per the sensitivity pattern of the organism and the infant was discharged.

## Case presentation

A term male infant (38 weeks), with birth weight of 2880 g (appropriate for gestational age) presented in out-patient department on day 4 of post-natal life with the complain of decrease acceptance of breast feeding, fast respiration and lethargy from day 1. The infant was born to 28-year old, second gravida mother via normal vaginal delivery with uneventful antenatal and natal course. The infant was on exclusive breast feeding. The clinical examination of the infant showed admission weight 2740 g, respiratory rate 72/min, heart rate 168 beats/min, capillary refill time of 4 s, audible grunting with subcostal and intercostal retraction, nasal flaring, mottling, abdominal distension, normal heart sounds and Downey’s score of 5/10. The infant was started on nasal Bubble CPAP (Continuous positive airway pressure) for providing support to respiratory system and was evaluated with chest radiograph and sepsis screen. The chest X-ray showed homogenous opacity in right lower and right middle lobe suggesting pneumonia. The infant was started on intravenous fluid and intravenous antibiotics (piperacillin-tazobactam and Amikacin). The infant was hypotensive (mean blood pressure—34 mmHg), hence was started on vasopressors. The sepsis screen revealed total leucocyte count of 4380/μl (leucopenia), absolute neutrophil count 976/μl (moderate neutropenia), platelet count of 35,000/μl (severe thrombocytopenia), Immature neutrophil/Total neutrophil (I/T) ratio of 0.36, micro ESR 18 mm in 1st h, and very high CRP (184 mg/l) (normal value < 10 mg/l). The peripheral blood film examination showed presence of toxic granulation in neutrophils. Blood gas analysis showed mixed acidosis with pH of 7.254, Pco_2_ 54 mmHg, bicarbonate 12.2 mmol/l and base deficit of—12.8 mmol/l. The infant developed respiratory failure after 5 h of admission for which infant was started on invasive ventilation for next 72 h. The infant required platelet transfusion for severe thrombocytopenia. The cerebrospinal fluid (CSF) analysis was suggestive of normal study (CSF sugar 74 mg/dl against blood sugar of 108 mg/dl, CSF protein 114 mg/dl (normal range 60–120 mg/dl) and cell count 04 cells/μl with 100% cells being lymphocytes). The blood culture sent from two different site using strict aseptic precaution with three swab techniques used before venipuncture, showed growth of *P. Rettgeri*. The organism was non-lactose fermenter on MacConkey agar, positive for citrate, urease, mannitol, inositol and rhamnose and negative for arabinose and sucrose. The antibiotic sensitivity pattern of the organism showed sensitivity to Meropenem, Imipenem, Chloramphenicol, and Cotrimoxazole, resistance to Ampicillin, Amikacin, Amoxycillin-clavulanate, Ampicillin, Cefazolin, Cefepime, Cefotaxime, Ceftazidime, Cefixime, Ceftriaxone, Cefoperazone-sulbactam, Ciprofloxacin, Colistin, Ertapenem, Gentamicin, Levofloxacin, Ofloxacin, Piperacillin + Tazobactam, Piperacillin, Ticarcillin, and Tobramycin. The CSF culture was not suggestive of any bacterial growth. The antibiotics were upgraded to Meropenem and gradually the clinical condition of the infant improved. Feeds were started on day 3 of admission and gradually increased to full feeds. The repeat blood culture sent after day 14 of intravenous Meropenem showed no growth. Head ultrasound showed normal study. The infant was discharged in good condition and was accepting breast feeding at the time of discharge.

## Discussion

Providencia are gram negative motile rods and are considered as opportunistic bacterial pathogen of clinical significance. The sources of Providencia are varied and with each species of Providencia having a preferred habitat. *P. alcalifaciens* and *P. rustigianii* have been isolated from the colon and feces, *P. stuartii* from wounds, urinary tract and respiratory tract and *P. heimbachae* from feces of penguins. The most commonly isolated Providencia in human being is *P. stuartii* and it leads to urinary tract infections and bacteremia in intensive care unit. The *P. rettgeri* has been isolated from poultry, feces of reptiles and amphibians and surface waters. It is rarely isolated from human feces and urinary tract. The bacterial urease causes alkalization of urine leading to encrustation of catheters. *P. rettgeri* has also been reported to cause wounds, burns and blood infection [3–7].

Proteeae comprises of three genera namely Proteus, Morganella and Providencia. Providencia is differentiated from other two members because of its ability of using citrate and fermenting d-mannitol [8]. The different strains of *Providencia* spp. have peritrichate flagella leading to its motility at 20–25°, but at temperature of 37° they are less motile. Providencia strains do not swarm, have fruity smell and requires neither niacin nor pantothenic acid for growth. The biochemical properties of Providencia family include positive for Indole production, Methyl Red, Citrate, Phenylalanine deaminase, growth in potassium cyanide, acid production from mannose, inositol, salicin, l-rhamnose, d-mannitol, adonitol, d-arabitol, and erythritol and negative for Voges-Proskauer reaction. *P. rettgeri* strains are genetically diverse when compared to other Providencia strains and are differentiated with other strains because of their property to ferment mannitol and degrade urea. Biogroups of *P. rettgeri* can be distinguished by their reactions in rhamnose, salicin and erythritol. Approximately 3/4th of isolates of *P. rettgeri* have the ability to acidify erythritol which is unique only to this species when compared to other members of Proteeae [8].

Infection caused by Providencia are rare and are mostly hospital acquired. The different infection caused by Providencia species are urinary tract infection, bacteremia, endocarditis, surgical site infection, soft tissue infection, brain abscess, meningitis, burn site infection, intravascular device infection, ocular infection, xanthogranulomatous pyelonephritis, peritonitis, intra-abdominal infection, and automatic implantable cardioverter-defibrillator pocket infection. The proposed pathogenesis of Providencia sepsis is ascending infection through the urinary tract and subsequent bacteremia leading to systemic infection and meningitis [3, 4, 9–13].

*Providencia rettgeri* is often resistant to multiple antibiotics like gentamicin, first-generation cephalosporins, nitrofurantoin, fosfomycin, tigecycline, polymyxins and ampicillin. Imipenem, amikacin and cefepime are the most active agents for more than 90% of the isolates [1, 14–16]. There is emergence of isolates that are carbapenemase producers carrying the New Delhi metallo-β-lactamase (NDM) gene bla _NDM-1_ [17]. Tshisevhe et al. reported outbreak of carbapenem-resistant Providencia rettgeri involving four patients in South Africa with isolates being resistant to Meropenem, Imipenem and Ertapenem [18].

Maiti et al. published case series of three patients having age of 3, 34 and 40 years with central nervous system infections caused by *P. rettgeri*. The organism was isolated from cerebrospinal fluid in two patients and from subdural empyema in one patient. The patients were treated successfully with antibiotics and were discharged [19]. There are no published case report till date that have reported Providencia rettgeri as cause of neonatal sepsis.

## Conclusion

Providencia are gram negative motile rods and are member of Enterobacteriaceae genus. They are differentiated from other genera of Proteeae on the basis of specific biochemical reactions. They lead to multiple disease in immunocompromised host. *Providencia rettgeri* is very rare organism causing neonatal sepsis and management includes using sensitive antibiotics.
